# Muscarinic ACh Receptors Contribute to Aversive Olfactory Learning in *Drosophila*

**DOI:** 10.1155/2015/658918

**Published:** 2015-08-25

**Authors:** Bryon Silva, Claudia Molina-Fernández, María Beatriz Ugalde, Eduardo I. Tognarelli, Cristian Angel, Jorge M. Campusano

**Affiliations:** Laboratorio Neurogenética de la Conducta, Facultad de Ciencias Biológicas, Pontificia Universidad Católica de Chile, Alameda 340, 8331150 Santiago, Chile

## Abstract

The most studied form of associative learning in *Drosophila* consists in pairing an odorant, the conditioned stimulus (CS), with an unconditioned stimulus (US). The timely arrival of the CS and US information to a specific *Drosophila* brain association region, the mushroom bodies (MB), can induce new olfactory memories. Thus, the MB is considered a coincidence detector. It has been shown that olfactory information is conveyed to the MB through cholinergic inputs that activate acetylcholine (ACh) receptors, while the US is encoded by biogenic amine (BA) systems. In recent years, we have advanced our understanding on the specific neural BA pathways and receptors involved in olfactory learning and memory. However, little information exists on the contribution of cholinergic receptors to this process. Here we evaluate for the first time the proposition that, as in mammals, muscarinic ACh receptors (mAChRs) contribute to memory formation in *Drosophila*. Our results show that pharmacological and genetic blockade of mAChRs in MB disrupts olfactory aversive memory in larvae. This effect is not explained by an alteration in the ability of animals to respond to odorants or to execute motor programs. These results show that mAChRs in MB contribute to generating olfactory memories in *Drosophila*.

## 1. Introduction

Different training protocols used in* Drosophila* have helped us advance our understanding on the cellular and genetic basis for learning and memory. One of the most studied and best understood is the associative learning of odors, where an odorant that has or does not has an intrinsic value for the animal (the CS) is paired with the US. Thus, the odorant acquires a new value for this animal. The type of memory generated depends on the quality of the US: while in some training protocols electric shock or aversive chemicals such as quinine or salt have been used as US to generate aversive memories [1], odors can also be paired with sugar to generate appetitive memories [2]. Behavioral and genetic studies have demonstrated that this associative learning depends on the integrity of the major neuropil in the fly brain, the MB, and their principal neurons, the Kenyon Cells (KCs) [3, 4]. Therefore, it has been accepted that the timely, coincident arrival of the information of the CS and the US to MB KCs is essential to generate new olfactory memories [3–6]. This is valid not only for adult flies but also in animals at the larval stage, as shown previously [7, 8].

The literature supports the idea that neurons containing and releasing BAs transmit the US information to the MB, both in adult flies and also in larvae [9, 10]. Remarkably, recent reports have advanced our knowledge on the neural aminergic pathways innervating the MB, the specific receptors activated, and some of the cellular events gated by amines in KCs that could underlie the generation of new memories both in larva and the adult fly [5, 11–13].

On the other hand, the CS is relayed to KCs through cholinergic inputs arising from the antennal lobe (AL) via the inner antennal cerebral tract [14]. This is consistent with the idea that ACh is the main excitatory neurotransmitter in the insect brain [15]. In mammals it is well known that ACh exerts its diverse actions by activation of the fast-acting ionotropic nicotinic receptors (nAChRs) [16] and also metabotropic muscarinic ACh receptors (mAChRs) [17]. Ten different genes encode the different subunits for* Drosophila* nAChRs and although the exact subunit composition of native fly neuronal nAChRs is not known, cell physiology experiments have helped us gain some insights on the functional properties of these channels. For instance, electrophysiological studies have shown that ACh activates *α*-bungarotoxin-sensitive nAChRs underlying fast excitatory synaptic currents in* Drosophila* brain neurons [18, 19]. Moreover, it has been recently shown in an* in vitro* preparation that the enhancement of the AL Projection Neuron-MB synapse depends on the activity of nAChRs [20]. Consistent with all these data, imaging studies have shown that activation of nAChRs induces an increase in intracellular calcium that mediates cellular plasticity [21].

On the other hand, one mAChR has been identified and cloned in* Drosophila *[22]. The* Drosophila* mAChR shows high sequence homology to the vertebrate M1-type mAChR and accordingly it was shown to increase the metabolism of membrane phospholipids when expressed in heterologous systems [23–25]. Interestingly, no information is available on the possibility that mAChRs are involved in olfactory processing in* Drosophila*, even though it has been shown that this receptor is highly expressed in MB [23, 25]. In our lab we have generated a new protocol to induce olfactory aversive memories in* Drosophila* larvae, based on the protocol presented and discussed in Gerber et al., 2010 [26]. By using this protocol we show for the first time that mAChRs expressed in* Drosophila* MB contribute to larval olfactory aversive learning and memory.

## 2. Materials and Methods

### 2.1. Fly Strains

Flies of the* w* ^1118^ (IsoCJ1) strain were used as control. This is an isogenized Canton-S strain carrying the* w* ^1118^ mutation [27, 28]. Flies bearing the OK107-GAL4 transgene were crossed to animals that contain the RNAi targeting the* Drosophila* mAChR (RNAi^mAChR^) under the control of UAS (line # 27571 obtained from the Bloomington* Drosophila* Stock Center, Indiana, USA), to direct the expression of this RNAi to MB. On the other hand, flies containing the mAChR-Gal4 element (obtained from the Vienna* Drosophila* Resource Center, Vienna, Austria, line # 201245) were crossed to transgenic animals containing the UAS-eGFP gene (part of the Campusano Lab fly stock, originally line #5431, Bloomington* Drosophila* Stock Center, Indiana, USA) to visualize cytosolic eGFP in the expression pattern of mAChR. In some experiments, we also used GH146-QF, QUAS-Tomato flies (line # 30037, Bloomington* Drosophila* Stock Center, Indiana, USA). In different experiments, mutants for the* Dunce* or* Rutabaga* proteins were used (dnc^1^ and rut^2080^, resp.). Flies were raised at 19°C, in a 12/12 hour light/dark cycle to decrease the expression of Gal4-driven genes and were brought to 25°C one day before the beginning of any experiment.

### 2.2. Protocol for Olfactory Learning and Memory in Larvae

**Figure 1:**
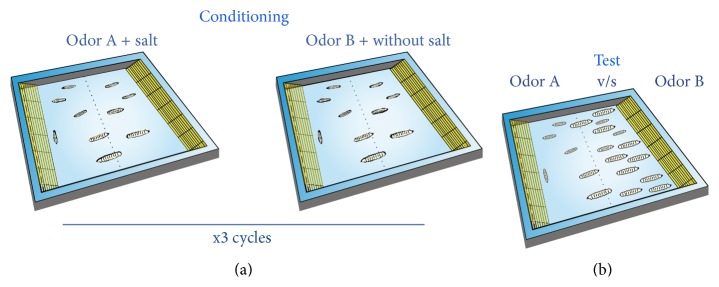
Schematic of the protocol used for generating aversive olfactory memories in* Drosophila* larvae. Training and memory tests are carried out in square shape Petri dishes that in opposite sides have containers where odorants are located (represented in yellow) (a) Training: fifteen or more larvae are exposed for 3 min to only one odorant in a dish half-covered with agar supplemented with 2 M NaCl (Odor A + salt). Then animals are exposed to a second odorant in a different Petri plate half-covered with regular agar. This is one training cycle. This procedure is repeated two more times. Afterwards, memory test is carried out. (b) Memory test: to evaluate aversive olfactory memory, animals are placed in the centerline of a square plate, so they are equally exposed to the two odorants, each one in opposite containers. After three minutes, the number of larvae at both sides of the center area is counted.

A new protocol to generate olfactory aversive memories based on the protocol discussed in Gerber at al., 2010 [26], was used in our experiments. The main difference is that training and test plates were 100 mm noncompartmentalized square-shape Petri dishes (Sterilin, UK). Containers for odorants were placed at opposite sides of the plate. The general arrangement is shown in Figure 1. Square-shaped plates were half-covered with solidified agar (1%), which was supplemented or not with 2 M NaCl (the US). All procedures (training, memory assessment, and olfactory discrimination assays) were carried out in plates covered by a lid, which was perforated in the center (0.5 mm diameter holes) for good aeration.

The training protocol was briefly as follows: 15 or more larvae were placed in one salt-containing agar plate where they were exposed for 3 min to one odorant, the CS. Then, animals were rinsed in water and afterwards placed in a second training plate where they were exposed to the second odorant; this time the agar contained no salt. This was one training cycle. This procedure was repeated 2 more times and in between training cycles, animals were rinsed with water. A 1 min intertrial interval was used. To test for memory after training, larvae were placed at the center of the arena and exposed to the two odorants, placed in containers at opposite sides. The position of the animals with respect to the center of the arena was recorded after 3 min.

This entire procedure was carried out a second time as explained, but inverting the use of odorants so that the odorant that was not paired with salt in the first experiment was the CS in this second experiment. This is called reciprocal training [26].

In all experiments a “no decision zone” was defined as a rectangular 7 mm × 10 mm area in the center of the memory test dish. Animals standing in this zone are not included in further data calculation. Only experiments where at least 13 larvae express a decision are included in this work.

To control for naïve responses at different odorants ratio (see Figure 1) the procedure used was the same as explained above when testing for memory formation, and data was expressed as “preference index.” Preference for a given odorant was calculated according to the following formula:

(1) $$\begin{array}{c} {Preference}_{A\;\;over\;\;B}=\frac{\left({\#}_{\text{A}}-{\#}_{\text{B}}\right)}{{\#}_{\text{Total}}}*100, \end{array}$$

which calculates the number of larvae in side close to odorant A minus the number of animals in side close to odorant B, divided by the total number of larvae. This figure represents the number of larvae preferring odorant A over B, as a percentage of the total number of larvae used in a given experiment.

After training, the memory generated was expressed as performance index, calculated according to the following formula:

(2) Performance  Index=PreferenceA+  over  B−PreferenceB+  over  A∗1002,

where “A+ over B” indicates that the odorant A was associated with salt, while “B+ over A” indicates that the odorant B was associated with salt. As the formula shows, the result indicates the number of larvae that learned the association between CS and US.

Odorants used in learning and memory experiments were ethyl acetate, EA, and n-amyl acetate, AA (1 : 10 and 1 : 100 dilution, resp., in paraffin oil). All these chemicals were obtained from Sigma-Aldrich (St Louis, MO). All behavioral experiments were carried out at the Campusano Lab Fly Room, maintained at 25°C, ~50% humidity, under constant illumination.

Memory performance was assessed at different time points after training (0, 5, 15, 30, and 45 min), with independent groups of animals for each time point: animals were trained and then memory was evaluated at a given time point. After this evaluation, animals were discarded.

To assess olfactory acuity, at least twenty larvae were exposed to one of the odorants in one side of the plate versus the vehicle (paraffin oil) in the other side. Three minutes later, the number of larvae in the odorant and in the vehicle sides was recorded. The same procedure was carried out for the other odorant using independent animal groups. Preference of larvae for odorants was calculated as explained above and preference indexes were all positive, as these are attractive odorants for larvae [29]. These data are presented as Supplementary Figures S1 and S2 (see Supplementary Material available online at http://dx.doi.org/10.1155/2015/658918).

### 2.3. Evaluation of Larva Locomotion

Experiments were carried out as indicated in [30]. Briefly, the movement of single third instar larvae was recorded for 140 secs (Olympus Digital Camera). To avoid the potential influence of external or visual cues, the recordings were carried out under constant illumination in a closed box. Motor behavior was analyzed using an automated tracking system (Image-Pro Plus 6.0 software; Media Cybernetics Inc, Rockville, MD, USA) and is expressed as distance covered by the animal (in mm) [30].

### 2.4. Atropine Treatment

Whenever required, animals were trained in presence of agar supplemented with atropine, a well-known antagonist of mAChRs. Different concentrations of atropine were used (10 nM, 1 *μ*M, and 100 *μ*M).

It is very difficult to assess the amount of atropine reaching the brain of larvae during our experiments. However, given the fact that we observe defects in olfactory memory (see Section 3) it is very likely that these effects are caused by this drug once it has reached the larval central nervous system (CNS). As an indirect method to estimate the amount of drug entering the larvae, we carried out experiments where agar was supplemented with a food colorant, tartrazine, 0.03 mg/mL (Comercial Cherry Ltda, Chile). Twenty animals were exposed to this colorant-supplemented agar for 1 h, in the same conditions used in the memory training experiments. Afterwards, larvae are rinsed with water and homogenized. After a centrifugation at 4000 rpm, colorant concentration is measured in the supernatant. Data obtained suggests that colorant reaches a concentration inside larvae of 0.21 ± 0.08 *μ*g/mL/larvae (*n* = 315 larvae).

### 2.5. Expression of mAChR in Larvae

Animals obtained from the mating of flies containing the mAChR-Gal4 and the UAS-eGFP genetic elements were crossed to recombinant animals bearing the GH146-QF, QUAS-Tomato transgenes. Only animals obtained from this cross that were both positive for GFP and Tomato fluorescence were imaged. Larvae were predissected in phosphate-buffered saline (PBS). The brains still attached to the body wall were fixed for 30 min in PBS containing 4% paraformaldehyde and subsequently rinsed in PBT (phosphate buffer containing 0.3% Triton X-100). Brains were mounted with Slowfade Gold Antifade Reagent with DAPI (Life Technologies, US) and visualized under a confocal spectral microscope Nikon Eclipse C2 using 20x, 40x, and 60x objectives, at a resolution of 1024 × 1024 pixels and a Z-step of 0.5 microns. The software used to process and display microphotographs was ImageJ.

### 2.6. Bioethical and Biosafety Issues

All experimental procedures were approved by the Bioethical and Biosafety Committee of the Facultad de Ciencias Biológicas, Pontificia Universidad Católica de Chile, and were conducted in accordance with the guidelines of the National Fund for Scientific and Technological Research (FONDECYT) and the Servicio Agrícola y Ganadero de Chile (SAG).

## 3. Results

### 3.1. Finding the Experimental Conditions for Aversive Olfactory Learning in* Drosophila* Larvae

**Figure 2:**
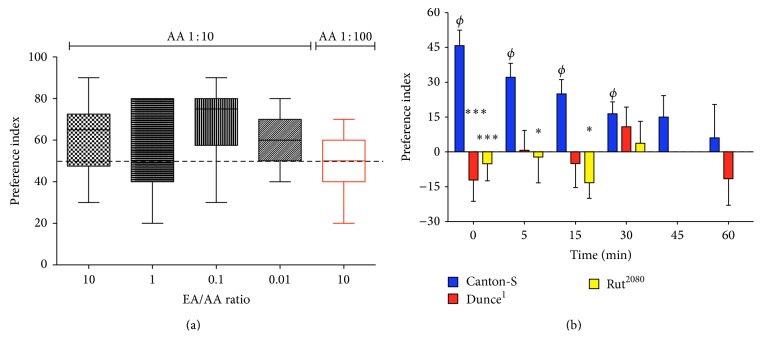
Establishing the conditions for larval aversive olfactory learning and memory. (a) Example of preference for AA over EA observed in larvae exposed to different ratios of EA/AA odorant dilutions. Odorant ratios induce a preference that is 60% or above, when the dilution of AA is set to 1 : 10 and the EA dilution is modified to obtain the indicated EA/AA dilution ratios (in black). On the other hand, when modifying the dilutions of both odorants to obtain an EA/AA ratio of 10 (in red), it leads to an equilibrated preference response (50% + 4.7%) that is different from the other dilutions shown. This data argues in favor of the idea that to control for responses to odorant dilutions is necessary for a balanced response of animals exposed to these stimuli. (b) Three training cycles induce a robust olfactory memory that lasts at least 30 min in larvae. Animals were subjected to a reciprocal training: larvae were exposed to one odorant in presence of salt and then to a second odorant that was not associated with salt. This training cycle was repeated two more times. Afterwards, animals were placed for 3 min in the test plate where the two odorants are present. The number of larvae in the conditioned and nonconditioned side of the chamber was recorded at different time points. Data show that control animals form an aversive memory, while two animals expressing a mutation for the cAMP signaling cascade (dunce^1^ and rut^2080^) do not. Each data presented (in a and b) was obtained from at least 10 different experiments, each one consisting of 15 or more larvae, so that the minimum amount of animals for any data point was 174 and 169 larvae in (a) and (b), respectively. *∗*, *∗∗∗* indicate *P* < 0.05 and *P* < 0.001, as compared to data obtained in control animals at the same time point (two-way ANOVA followed by Bonferroni multiple comparison post hoc test). *ϕ* indicates data different from zero in control animals (*P* < 0.05, Wilcoxon signed rank test). None of the values obtained in mutants are different from zero.

*Drosophila *larvae can be trained to avoid odors associated with different aversive stimuli, including electric shocks or chemicals such as quinine or salt. Reciprocal training using two different odorants diminishes the variability associated, among other factors, with the naïve preference expressed by an animal for one of the odorants.  Figure 2(a) shows a typical behavioral response observed in control larvae when exposed to the two odorants EA and AA. Data expressed as preference when animals are exposed to different ratios of EA to AA dilutions show a median close to or above 60% for all experimental conditions (boxes in black) and a big variability. These data were obtained modifying only the dilution of EA while AA was used at a 1 : 10 dilution and reflects how important it is to control for the naïve response of larvae to odorants, as to find dilutions that lead to an equal distribution of animals when in presence of the two odorants. The last data shown (Figure 2(a), box in red) present the naïve response of larvae exposed to EA (1 : 10 dilution) and AA (1 : 100 dilution). In this condition, preference expressed by animals for odorants is 50 ± 4.7%. These are the odorant dilutions used in the rest of this work.

It has been previously shown that the duration of the memory generated in larvae depends on different factors including the learning protocol used (e.g., how many training cycles are used) or the quality and intensity of the US. We have explored some of these issues and have found that using three training cycles leads to memory that lasts 30–45 min. By this time period, the memory performance decreases to a level where no preference for odorants is detected (Figure 2(b)). Moreover, our data show that this olfactory memory depends on cAMP signaling, since it is not observed in the cAMP phosphodiesterase mutant* Dunce* or in the calcium-calmodulin-dependent adenylate cyclase mutant* Rutabaga* (Figure 2(b)). Altogether these data show that aversive olfactory memory can be generated in* Drosophila* larvae, and as expected for this type of associative memory, it is short-lived and depends on cAMP, consistent with previous reports [5, 31, 32].

### 3.2. mAChR Contribution to Aversive Memory in* Drosophila* Larvae

Once the conditions to generate olfactory aversive memory were obtained, we decided to assess the contribution of mAChRs to this associative behavior.

**Figure 3:**
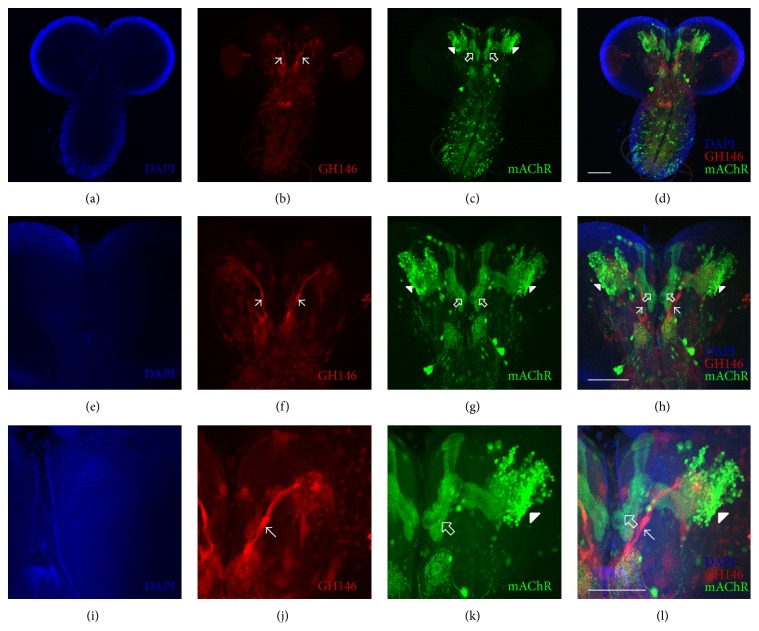
Expression pattern of mAChR in the larval brain. Animals expressing GFP in the pattern of the mAChR-Gal4 line were mated with flies expressing GH146-QF, QUAS-Tomato. (a)–(d) are photomicrographs obtained at a magnification of 20x; ((e)–(h)) images at 40x; ((i)–(l)) at 60x. (a), (e), and (i) present DAPI fluorescence in blue; (b), (f), and (j) show red-tomato fluorescence under the expression pattern of the AL Projection Neurons; ((c), (g), and (k)) GFP expression under the control of the mAChR-Gal4; (d), (h), and (l) are overlays of the blue, red, and green images to the left. Cell bodies and processes expressing GFP under the control of mAChR expression pattern are observed in the ventral nerve cord and the MB region, particularly in the calyx region (indicated by white arrowheads) and the larval MB lobes (shown by white empty arrows). AL Projection Neurons are shown in (b), (f), (j), (h), and (l) by white arrows. Then high expression of mAChR is detected in the MB region. Microphotographs obtained from representative experiment. Scale bars indicate 50 microns.

First, we evaluated the expression of mAChRs in larvae. Flies expressing eGFP under the control of mAChR-Gal4 were mated with animals containing the GH146-QF, QUAS-Tomato transgenes. Thus, in these animals eGFP is expressed according to the expression pattern of the receptor, while in red it is possible to identify the AL Projection Neurons and their connection with the MB Kenyon Cells. As shown in Figure 3, mAChR is expressed at some level throughout the entire larval CNS, but it is possible to clearly observe cell bodies in the ventral nerve cord and also in the larval brain, in and surrounding the MB region. Higher magnification microphotographs show that mAChR is localized in the calyx and larval MB lobes (Figures 3(i)–3(l)). Little expression is detected in the antennal lobe. This data shows that mAChR is expressed in the larval MB.

**Figure 4:**
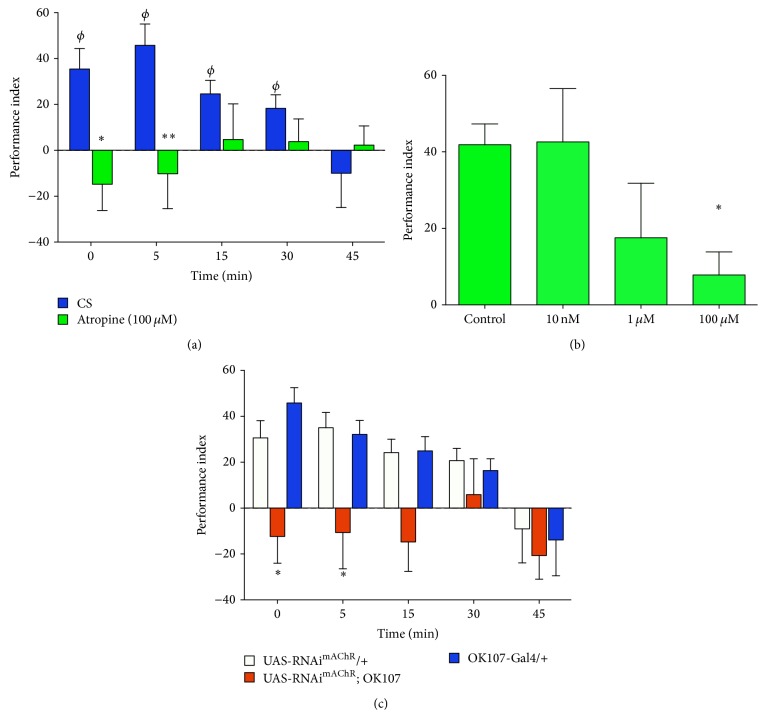
Genetic and pharmacological blockade of mAChRs disrupt aversive olfactory memory. (a) Flies exposed to the mAChR antagonist atropine (100 *μ*M, green bars) are not able to form the aversive olfactory memory as compared to control animals (blue bars). Each data presented was obtained from at least 16 different experiments, each one consisting of 15 or more larvae, so that the minimum amount of animals included in any data point was 317 larvae. *∗*, *∗∗* indicate *P* < 0.05 and *P* < 0.01, as compared to data in control animals at the same time point (two-way ANOVA followed by Bonferroni multiple comparison post hoc test). *ϕ* indicates data different from zero in control animals (*P* < 0.05, Wilcoxon signed rank test). None of the values obtained in RNAi expressing animals was different from zero. (b) Atropine effect is dose-dependent: while no effect on memory is observed in flies exposed to an antagonist concentration of 10 nM, a strong reduction in aversive memory is observed at 100 *μ*M. Partial reduction although with big variability is observed in flies exposed to 1 *μ*M atropine. Values shown for memory performance correspond to data obtained 5 min after training, when training and memory test were carried out in presence of indicated concentrations of the drug. Each type of data was obtained from at least 10 experiments, each one including 15 or more larvae. The minimum amount of larvae in any data point was 151 animals. *∗* indicates *P* < 0.05 as compared to control (one-way ANOVA followed by Dunn's multiple comparison test). (c) Expression of an RNAi for mAChR (RNAi^mAChR^) in MB disrupts olfactory memory formation. Each data presented was obtained from at least 15 different experiments, each one consisting of 15 or more larvae, so that the minimum amount of animals included in any data point shown was 299 larvae. *∗* indicates *P* < 0.05 as compared to data in genetic control animals at the same time point (two-way ANOVA followed by Bonferroni multiple comparison post hoc test). Genetic controls show values for performance index different from zero at 0, 5, and 15 min (*P* < 0.05, Wilcoxon signed rank test). None of the values obtained in RNAi expressing animals was different from zero.

Two different approaches were used to evaluate the contribution of mAChRs to aversive olfactory memory: one pharmacological and one genetic. For the first one,* Drosophila* larvae were exposed to atropine (100 *μ*M), a well-known mAChR antagonist, while being trained up to the memory assays. Data obtained show that this pharmacological treatment abolishes the ability of larvae to generate olfactory aversive memory (Figure 4(a)). On the other hand, our data show that the effect of atropine is dose-dependent (Figure 4(b)). Since treatment with 100 *μ*M atropine does not affect the olfactory acuity of larvae (Figure S1), these results suggest that mAChRs contribute to the generation of olfactory aversive memory. It has been shown that, as in adult flies, larval MB contribute to the expression of motor programs [30, 33]. Thus, it is important to control for locomotion in animals treated with atropine. Experiments carried out in animals exposed to atropine 100 *μ*M demonstrate that this manipulation does not affect motor output in these animals (larvae exposed to atropine covered 113.1 ± 14.4 mm versus 95.0 ± 8.5 mm in control animals, *n* = 12 and 10 larvae resp., *t*-test, *P* > 0.05). Thus, all these data support the proposition that mAChRs are contributing to the generation of an aversive olfactory memory in* Drosophila* larvae, but it does not address where mAChRs are acting to modulate this memory.

To evaluate this issue, we turned to a genetic approach. By using the Gal4-UAS technique, we expressed an RNAi for mAChR (RNAi^mAChR^) in the larval brain region associated with olfactory memories, the MB. Animals were trained as explained above and memory performance was assessed as indicated elsewhere. Results obtained show that aversive memories are not formed in animals expressing the RNAi^mAChR^ in MB (Figure 4(a)). Experiments carried out in animals expressing the RNAi^mAChR^ in MB show no effect of this genetic manipulation on larval locomotion when compared to control animals (112.5 ± 8.5 mm covered by animals expressing the RNAi^mAChR^ in MB, as compared to 90.5 ± 6.8 mm and 116.1 ± 7.9 mm in OK107-Gal4/+ and UAS-RNAi^mAChR^ /+ animals, resp., *n* = 16, 10, and 10 animals, resp., *P* > 0.05 ANOVA followed by Tukey post-test). Altogether these data show that mAChRs are required in MB for the generation of an aversive olfactory memory.

## 4. Discussion

Different proteins and molecules have been associated with the formation of memory in different systems. Remarkably, some of the key contributors to learning and memory are highly conserved from arthropods to mammals. This makes it possible to study some of the basic principles underlying learning and memory in invertebrates, knowledge that can be later extrapolated to more complex systems [34, 35].

### 4.1. The Associative Olfactory Training in* Drosophila* Larvae

In our lab we try to elucidate the contribution of receptors and neural systems to complex behaviors including olfactory learning and memory. In order to progress on this subject, we established a protocol for the formation of associative aversive olfactory memory in* Drosophila* larvae. It has been suggested that the larva is as good as a model system to elucidate some of the cellular and molecular conditionings underlying olfactory learning and memory in flies, as compared to adult flies. The larva is considerably simpler in number of cells and overall organization of the olfactory system [7, 8, 36], which is one of the reasons we and others use it as an animal model to get new insights on the cellular and molecular mechanisms responsible for the generation of new memories.

The reciprocal training protocol we regularly use in our experiments is aimed at getting a robust, reproducible memory of odors that is thought to be independent of the odorant dilutions used for training and/or memory testing, as in different set of experiments the US-paired odorant is switched [26]. This is different from training protocols where only one odorant is associated with an US [32, 37]. However, our data show that even when using the reciprocal training protocol it is necessary to establish the adequate experimental conditions leading to an equilibrated distribution of animals exposed to the odorants in the test chamber before any training. In fact, two different experiments carried out with EA/AA dilution ratios of 10 lead to a different naïve preference: when this ratio is obtained starting from a 1 : 10 AA dilution, preference observed is above 60%; when a 1 : 100 AA dilution is used to prepare this dilution ratio, the preference observed is about 50%. This data suggests that it is important to control for naïve preference of animals for the odorants to be used in olfactory learning and memory experiments, as this complex behavior depends on the ability of animals to adequately sense and respond to odorant stimuli. Other factors that could also affect the performance of animals in this associative behavior include the presence of drugs (in our case atropine) or the US (i.e., salt in our experiment). All these factors have been controlled in our experiments (data shown as Supplementary Figures  S1 and S2) to make sure the results are indeed explained by the ability of animals to generate new memories.

### 4.2. mAChRs in* Drosophila* Aversive Learning

The existence of one G-protein coupled metabotropic muscarinic ACh receptor (mAChR, aka mAChR-A) has been shown in* Drosophila *[22]. This mAChR shows high sequence homologyto vertebrate M1-type mAChRs and as expected induces the activation of PLC to modulate membrane phospholipids in heterologous systems [22–24]. Recently a second mAChR was identified (a.k.a. mAChR-B [38]). This second putative mAChR shows several differences in its amino acid sequence and pharmacological and physiological properties with all previously described vertebrate and invertebrate mAChRs, including the fact that it is not activated by muscarine or blocked by atropine or scopolamine, two well-known mAChR antagonists [38]. Thus, it is not clear whether this is actually a mAChR. For all these reasons we focused our work only on the mAChR-A.

Expression studies have shown that the mAChR is highly expressed in the adult MB and AL [23, 25]. Information obtained from high-throughput expression studies indicates that this receptor is also expressed in the larval CNS [39], although up to now there was no information on the expression of this receptor in specific larval brain regions. Our data show for the first time that the mAChR is expressed in somas and processes in the ventral nerve cord and the larval MB region, specifically in the calyx and larval MB lobes, positioning this receptor in the right place to modulate olfactory learning.

Two different but complementary approaches were used to assess the contribution of mAChRs to olfactory aversive learning in larvae. In one hand, animals were trained and tested in presence of atropine, a well-known antagonist for mAChRs. Data obtained show that these animals are unable to form an aversive olfactory memory, which suggests that mAChRs are required for memory formation. This approach does not speak of the site where mAChRs are acting to modulate memory formation, and therefore several situations could explain this result. Since cholinergic neurons convey the information of the CS to the MB, it is possible that mAChRs are presynaptically located in the AL Projection Neuron-MB synapse to modulate ACh release in the MB region, similar to what has already been suggested for nAChRs in an* in vitro* AL-MB synapse preparation [20]. mAChRs could also be located in the aminergic terminals responsible for sending the information of the US to the MB, modulating this synapse. The modulation of the release of amines by cholinergic ligands is a possibility we have recently shown in an* in vitro* fly brain preparation [40]. It is also possible that mAChRs expressed in the MB neurons directly modulate the activity of these cells to induce memory formation. Since our expression studies support this proposition, we turned to a genetic approach to assess this last possibility. Remarkably, the specific expression of an RNAi^mAChR^ in MB fully inhibited the formation of new aversive olfactory memory in larvae. Altogether, these data demonstrate for the first time that mAChRs expressed in MB are required for the generation of aversive memory in* Drosophila* larvae.

The contribution of mAChRs in olfactory memory is something already established in other systems. For instance, it has been previously shown that mAChRs contribute to olfactory memories in honeybees [41, 42]. Interestingly, these data support the idea that the muscarinic receptors are only required for olfactory memory retrieval, not acquisition. Moreover, the effect of mAChRs on olfactory memory in honeybees depends specifically on the MB *α*-lobe [43]. We do not know whether mAChRs are required for specific memory phases or processes in* Drosophila* or if as in bees mAChRs are required in specific larval MB regions, but these are issues that we are currently evaluating.

On the other hand, it has been shown that the administration of scopolamine, a nonselective antagonist for M1–M5 vertebrate mAChRs, decreases different types of memory in mammals [44–46]. Moreover, data obtained in mice expressing a mutation for the M1-type mAChR show defects on memory acquisition and consolidation [47]. Remarkably, rats treated with scopolamine in the prelimbic cortex show deficient olfactory memory [48]. These data show that mAChRs are important contributors in the generation of memories, particularly olfactory memory, in mammals as it is in insects. Our data contribute to the understanding of the molecular underpinnings of memory formation in* Drosophila* but further support the proposition that regardless of obvious anatomical differences, the key contributors to complex phenomenon including olfactory learning and memory are conserved from arthropods to mammals.

## Supplementary Material

Fig S1. Olfactory acuity and discrimination are not altered by salt or salt + atropine treatments in control animals. A, B, Behavior of larvae was recorded in a test plate where one odorant was in one side (A, Amyl acetate; B, Ethyl acetate) while the vehicle (paraffin oil) was in the other side. Test plates were half-filled with agarose (control group), with agarose supplemented with 2M NaCl (Salt group) or with salt and 100 μM atropine (Salt + Atropine group). No statistical differences (n.s.) were observed in the Preference Index calculated for the larvae in presence of odorants versus vehicle, in any experimental condition (P>0.05, one way ANOVA). C. Larvae exposed to the two odorants in a control agar test plate, or in test plates containing salt or salt+ atropine, showed no statistical difference in their calculated Preference Index, as compared to control condition (P>0.05, one way ANOVA). Each data shown in A–C correspond to a minimum of 15 experiments, each one including at least 15 larvae, so that the smallest number of larvae included in any individual data in this entire figure is 283 animals.
Fig S2. Olfactory acuity and discrimination are not altered in animals expressing the RNAi for mAChR. Animals used in these studies were expressing the RNAi for mAChR in MB (UAS-RNAi^mAChR^; OK107, orange bars) and their genetic controls (UAS-RNAi^mAChR^/+, white bars; and OK107-Gal4/+, rose bars). A, B, Behavior of larvae was recorded in a test plate where one odorant was in one side while the vehicle (paraffin oil) was in the other side (A, Amyl acetate; B, Ethyl acetate). Test plates were half-filled with agarose. No statistical differences (n.s.) were observed in the Preference Index calculated for the larvae in presence of odorants versus vehicle (P>0.05, one way ANOVA). C. Larvae of UAS-RNAi^mAChR^; OK107 showed no statistical difference in their calculated Preference Index as compared to genetic controls, when exposed to the two odorants in an agar test plate (P>0.05, one way ANOVA). Each data shown in A–C correspond to a minimum of 15 experiments, each one including at least 15 larvae, so that the smallest number of larvae included in any individual data in this entire figure is 301 animals.
