# Supplementary material for: Muscarinic ACh Receptors Contribute to Aversive Olfactory Learning in Drosophila
Source: Neural Plast. 2015 Aug 25;2015:658918. doi: 10.1155/2015/658918 (PMC4562076; doi:10.1155/2015/658918)
Supplement: Supplementary file 1 — Fig S1. Olfactory acuity and discrimination are not altered by salt or salt + atropine treatments in control animals. A, B, Behavior of larvae was recorded in a test plate where one odorant was in one side (A, Amyl acetate; B, Ethyl acetate) while the vehicle (paraffin oil) was in the other side. Test plates were half-filled with agarose (control group), with agarose supplemented with 2M NaCl (Salt group) or with salt and 100 μM atropine (Salt + Atropine group). No statistical differences (n.s.) were observed in the Preference Index calculated for the larvae in presence of odorants versus vehicle, in any experimental condition (P>0.05, one way ANOVA). C. Larvae exposed to the two odorants in a control agar test plate, or in test plates containing salt or salt+ atropine, showed no statistical difference in their calculated Preference Index, as compared to control condition (P>0.05, one way ANOVA). Each data shown in A–C correspond to a minimum of 15 experiments, each one including at least 15 larvae, so that the smallest number of larvae included in any individual data in this entire figure is 283 animals. Fig S2. Olfactory acuity and discrimination are not altered in animals expressing the RNAi for mAChR. Animals used in these studies were expressing the RNAi for mAChR in MB (UAS-RNAimAChR; OK107, orange bars) and their genetic controls (UAS-RNAimAChR/+, white bars; and OK107-Gal4/+, rose bars). A, B, Behavior of larvae was recorded in a test plate where one odorant was in one side while the vehicle (paraffin oil) was in the other side (A, Amyl acetate; B, Ethyl acetate). Test plates were half-filled with agarose. No statistical differences (n.s.) were observed in the Preference Index calculated for the larvae in presence of odorants versus vehicle (P>0.05, one way ANOVA). C. Larvae of UAS-RNAimAChR; OK107 showed no statistical difference in their calculated Preference Index as compared to genetic controls, when exposed to the two odorants in an agar test plate (P>0 [file 658918.f1.pdf]

Supplementary Figures.

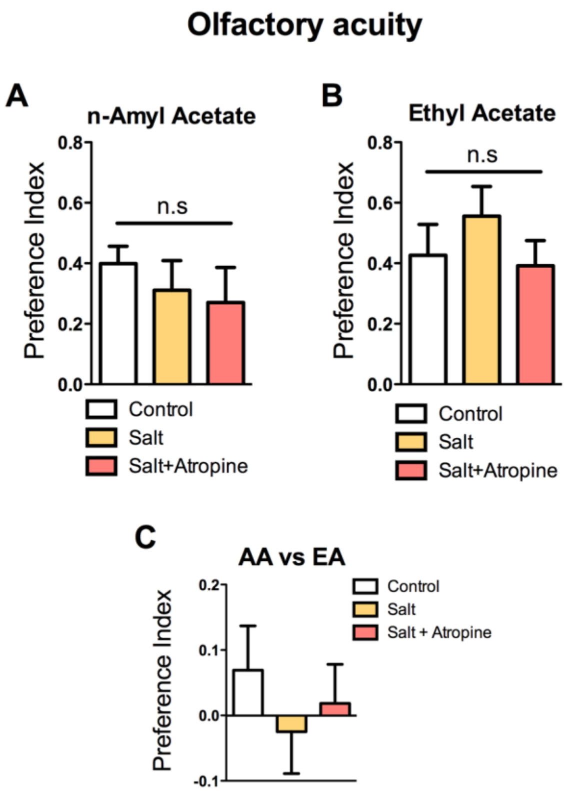

Fig S1. Olfactory acuity and discrimination are not altered by salt or salt + atropine treatments in control animals. A, B, Behavior of larvae was recorded in a test plate where one odorant was in one side (A, Amyl acetate; B, Ethyl acetate) while the vehicle (paraffin oil) was in the other side. Test plates were half-filled with agarose (control group), with agarose supplemented with 2M NaCl (Salt group) or with salt and 100 µM atropine (Salt + Atropine group). No statistical differences (n.s.) were observed in the Preference Index calculated for the larvae in presence of odorants versus vehicle, in any experimental condition ($p>0.05$, one way ANOVA). C. Larvae exposed to the two odorants in a control agar test plate, or in test plates containing salt or salt+ atropine, showed no statistical difference in their calculated Preference Index, as compared to control condition ($p>0.05$, one way ANOVA). Each data shown in A-C correspond to a minimum of 15 experiments, each one including at least 15 larvae, so that the smallest number of larvae included in any individual data in this entire figure is 283 animals.

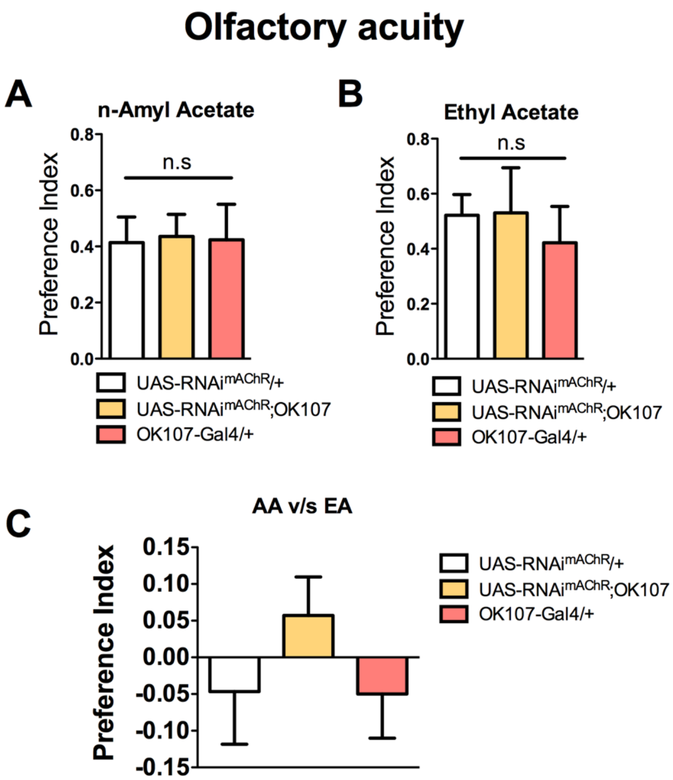

Fig S2. Olfactory acuity and discrimination are not altered in animals expressing the RNAi for mAChR. Animals used in these studies were expressing the RNAi for mAChR in MB (UAS-RNAi^{mAChR};OK107, orange bars) and their genetic controls (UAS-RNAi^{mAChR}/+,white bars; and OK107-Gal4/+, rose bars). A, B, Behavior of larvae was recorded in a test plate where one odorant was in one side while the vehicle (paraffin oil) was in the other side (A, Amyl acetate; B, Ethyl acetate). Test plates were half-filled with agarose. No statistical differences (n.s.) were observed in the Preference Index calculated for the larvae in presence of odorants versus vehicle ($p>0.05$, one way ANOVA). C. Larvae of UAS-RNAi^{mAChR};OK107 showed no statistical difference in their calculated Preference Index as compared to genetic controls, when exposed to the two odorants in an agar test plate ($p>0.05$, one way ANOVA). Each data shown in A-C correspond to a minimum of 15 experiments, each one including at least 15 larvae, so that the smallest number of larvae included in any individual data in this entire figure is 301 animals.
